# Epidemiological Trends in Mesothelioma Mortality in Colombia (1997–2022): A Retrospective National Study

**DOI:** 10.3390/ijerph22050787

**Published:** 2025-05-16

**Authors:** Luisa F. Moyano-Ariza, Guillermo Villamizar, Giana Henríquez-Mendoza, Arthur Frank, Gabriel Camero

**Affiliations:** 1 Faculty of Engineering, University of Cartagena, Cartagena 110311, Colombia; lfmoyanoa@unal.edu.co; 2 Epidemiology and Evaluation in Public Health Research Group, National University of Colombia, Bogotá 111321, Colombia; 3 Colombia Asbestos Free Foundation, Bogotá 111211, Colombia; 4 National Cancer Institute, Bogotá 111511-110411001, Colombia; politicasymovilizacionsocial@cancer.gov.co; 5 Department of EOH, Drexel University School of Public Health, Philadelphia, PA 19104, USA; alf26@drexel.edu; 6 Colombian Red Cross—Cundinamarca-Bogotá Section, Emergencies, Disasters and Humanitarian Aid Research Group, Cundinamarca and Bogotá Red Cross, Bogotá 110211, Colombia; presidencia@cruzrojabogota.org.co

**Keywords:** mesothelioma, Colombia, asbestos exposure, mortality trends, public health, ICD-10, epidemiology, occupational disease

## Abstract

Background: Mesothelioma is a rare and aggressive cancer primarily caused by asbestos exposure. In Colombia, asbestos use began in 1942, but mortality surveillance remains limited. Long latency periods and poor documentation hinder public health action. Materials and Methods: A retrospective descriptive study was conducted using mortality data from 1997 to 2022 obtained from the National Administrative Department of Statistics (DANE), including all mesothelioma cases recorded under the five ICD-10 diagnostic categories (C45.0 to C45.9), covering all anatomical sites of first occurrence. Variables analyzed included sex, age, occupation, and place of residence. Mortality rates and trends were estimated using R, Excel, JoinPoint, and Minitab. Results: A total of 1539 mesothelioma deaths were recorded. Most occurred in men (65.1%) and in individuals over 60 years old (62.6%). Urban areas accounted for 92% of deaths. The most frequent diagnosis was unspecified mesothelioma (61.3%). Cities with the highest adjusted mortality rates per 100,000 inhabitants were Sibaté (38.36), Soacha (8.41), and Bogotá (1.89), aligning with historical exposure zones. Conclusions: Mesothelioma is still a preventable public health issue in Colombia, with sustained mortality affecting even working-age populations. The high rate of unspecified diagnoses and weak linkage between morbidity and mortality data underscore the need to strengthen diagnostic capacity, improve surveillance, and implement a national asbestos-related disease elimination strategy.

## 1. Introduction

Malignant mesothelioma is a rare and aggressive malignancy with high lethality and poor prognosis [1,2,3,4]. It has been described as a “manmade epidemic” resulting from widespread industrial use of asbestos over the past century [5]. While asbestos has been banned or restricted in many countries, legacy exposure continues to pose significant public health risks, especially in low- and middle-income settings [6].

In Colombia, industrial use of asbestos began in 1942 with the opening of the Eternit Colombiana plant in Sibaté, Cundinamarca [7]. Despite decades of widespread use in the construction and automotive industries, it was only in 2019 that the country enacted a complete ban under Law 1968 [8]. The long latency period of mesothelioma—often 30 to 40 years or more—means that new cases will continue to emerge for decades, even after the ban on asbestos. In the scientific literature, mesothelioma is widely regarded as a disease strongly associated with asbestos exposure. It is even considered a ‘signal tumor’ for asbestos, due to the strength and specificity of this relationship [9,10]. Although rare cases have been linked to other agents [11,12] or genetic susceptibility [13], asbestos remains the predominant cause worldwide. This monocausal understanding is supported by international evidence [14,15] and appears consistent with national mortality patterns observed in Colombia.

Although Colombia has made some progress in characterizing the burden of mesothelioma—particularly in recent years—significant gaps persist in national surveillance systems for mortality and morbidity. These include a high proportion of unspecified diagnoses, limited awareness among physicians about mesothelioma coding, and fragmentation across health information systems [16]. Moreover, women and populations exposed to environmental or para-occupational sources remain under-represented in official records, despite growing evidence of mesothelioma risk associated with non-occupational exposure routes [17].

Globally, organizations such as the World Health Organization and the International Labour Organization have urged countries to adopt national programs aimed at eliminating asbestos-related diseases [18]. In this context, mortality data are often used as a proxy for incidence, particularly in diseases like mesothelioma, which are characterized by long latency periods, short survival times, and limited access to early diagnosis in many settings.

This study presents the first nationwide retrospective analysis of mesothelioma (all sites) mortality in Colombia covering a 25-year period (1997–2022), using disaggregated official ICD-10-coded mortality microdata. While previous studies [16] have addressed the mesothelioma burden over shorter timeframes (2015–2020) using combined prevalence and mortality data, this is the first to examine long-term mortality trends at a national scale with detailed geographic and demographic resolution.

## 2. Materials and Methods

### 2.1. Study Design

This study is a retrospective, descriptive analysis of mesothelioma (all sites) mortality in Colombia over a 25-year period (1997–2022). The analysis was conducted using anonymized secondary data obtained from official national mortality records. It focused on all deaths attributed to malignant mesothelioma, as defined by the International Classification of Diseases, 10th Revision (ICD-10). The start year, 1997, corresponds to the implementation of ICD-10 in Colombia, which enabled the systematic coding of mesothelioma cases.

### 2.2. Data Sources

Mortality data were obtained from the Colombian National Administrative Department of Statistics (DANE), which compiles and publishes annual microdata on non-fetal mortality nationwide. The dataset included mesothelioma-related deaths classified under the five ICD-10 codes: C45.0 (mesothelioma of pleura), C45.1 (peritoneum), C45.2 (pericardium), C45.7 (other specified sites), and C45.9 (unspecified site). These five codes collectively represent all anatomical sites of first occurrence of mesothelioma as defined in the ICD-10 classification.

Additionally, morbidity data were retrieved from the Social Protection Health Information System (SISPRO), through the Individual Service Provision Records (RIPS), covering the period 2009–2022. Although mortality and morbidity records could not be directly cross-linked, their parallel analysis enabled the identification of potential inconsistencies in mesothelioma reporting.

### 2.3. Variables and Coding

The primary outcome variable was mortality due to mesothelioma. Variables included sex, age at death, place of residence and occurrence, ICD-10 diagnostic category, and occupation (available from 2008 onward). Age was grouped into five categories: 14–29, 30–44, 45–59, 60–74, and 75 years and older. For spatial analysis, administrative codes were used to classify deaths by department and municipality.

New derived variables were generated to standardize the data across the 25-year period. These included aggregated mesothelioma types, body site location, and a categorical occupational classification based on the national catalog published by the Colombian Ministry of Labor. Additionally, five-year age intervals were constructed from the raw age-at-death data in the mortality microdata. These intervals were used specifically for trend analysis and age-standardized rate calculations, while broader age groups were applied for descriptive purposes.

### 2.4. Statistical Analysis

Descriptive statistics were used to analyze absolute and relative frequencies by sex, age, diagnosis, and occupation. Mortality rates were calculated at national and city levels and were adjusted by age and sex using the direct method, with WHO population parameters as the standard. Population denominators were obtained from DANE population projections, which included adjustments for COVID-19-related demographic changes.

Trend analyses were performed using the JoinPoint Regression Program to assess annual percent changes (APC) for each type of mesothelioma. In addition, Minitab 19 was used to conduct median rate analysis, nested dispersion analyses, process control charts, and a binary decision tree. All datasets were cleaned and analyzed using Excel and RStudio 4.2.1.

### 2.5. Ethical Considerations

This research was conducted in accordance with Resolution 8430 of 1993 issued by the Colombian Ministry of Health, which establishes the scientific, technical, and administrative standards for health research in the country [19]. As this study used anonymized secondary data without any direct interaction with human subjects, it was classified as minimal risk and did not require approval from an ethics review board.

## 3. Results

Between 1997 and 2022, a total of 1539 deaths from malignant mesothelioma were reported in Colombia. During this 25-year period, the national population grew from 37.5 million to 51.6 million, reflecting an estimated 38% increase, with an average annual growth of approximately 500,000 inhabitants. Notably, both 2019 and 2020 recorded population increases exceeding one million.

**Figure 1:**
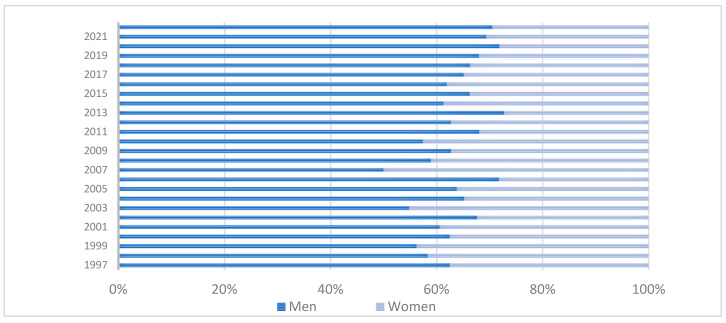
Percentage of deaths due to mesothelioma, according to sex, Colombia, 1997–2022. Source: DANE—National Administrative Department of Statistics.

Among the mesothelioma deaths recorded, 98% occurred in state capital cities, highlighting the urban concentration of mortality reporting. Regarding sex distribution, 65.1% occurred in men (n = 1002) and 34.9% in women (n = 537), resulting in a male-to-female mortality ratio of approximately 2:1 (Figure 1). This male predominance aligns with historical patterns of occupational asbestos exposure documented globally and in Latin America [9,20].

**Figure 2:**
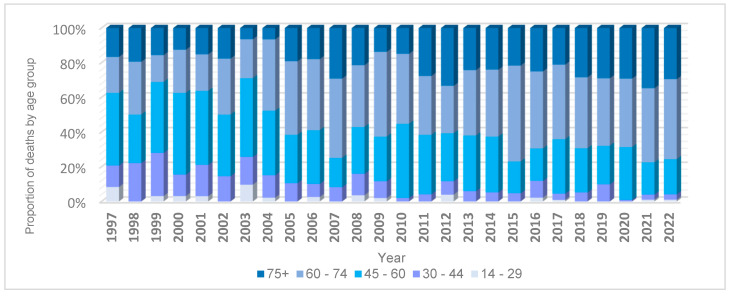
Percentage of deaths due to mesothelioma, by age group, Colombia, 1997–2022. Source: DANE—National Administrative Department of Statistics.

The percentage distribution of mesothelioma mortality by age group is presented in Figure 2. The majority of deaths (62.6%; n = 963) occurred in adults over 60 years of age. This was followed by the 45–59 age group, which accounted for 28.3% (n = 435) of all deaths. Individuals aged 30–44 represented 8.0% (n = 121), while only 1.3% (n = 20) of deaths occurred in individuals under 30 years old.

Mesothelioma deaths were also reported among children aged 5–14 in the years 1997, 2001, 2003, 2012, and 2016. Although rare, these cases are particularly concerning given the long latency period typically associated with the disease. Their occurrence raises concern about early-life exposure, possibly through environmental or para-occupational routes, such as handling contaminated materials brought home by family members or living near asbestos-contaminated sites [21,22]. Children may be more biologically vulnerable to asbestos exposure due to immature lung development, higher ventilation rates per body weight, and underdeveloped immune defenses. Some studies also suggest a higher lifetime risk of mesothelioma when exposure occurs during childhood, although findings are not entirely consistent [23,24]. In this context, the use of asbestos-contaminated talc in consumer products represents a particularly concerning and under-recognized pathway of early-life exposure [25].

Regarding mesothelioma-related mortality, 61.3% (n = 944) of the reported cases during the study period corresponded to mesothelioma of unspecified site (ICD-10: C459).

**Figure 3:**
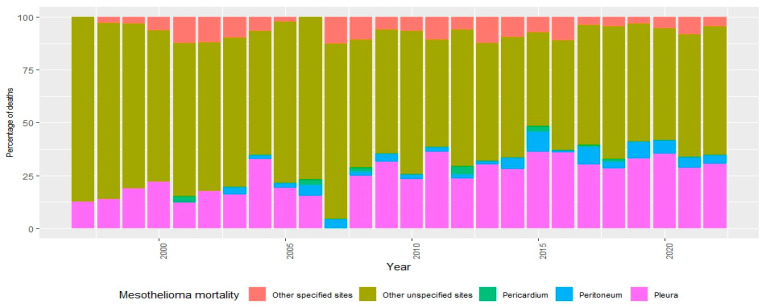
Percentage of deaths due to mesothelioma, by diagnosis, Colombia, 1997–2022. Source: DANE—National Administrative Department of Statistics.

This was followed by mesothelioma of the pleura (C450) with 27.6% (n = 424), mesothelioma of other specified sites (C457) with 6.0% (n = 101), mesothelioma of the peritoneum (C452) with 4.0% (n = 61), and mesothelioma of the pericardium (also C452) with 0.6% (n = 9) (Figure 3).

The predominance of unspecified diagnoses raises concerns about the quality and precision of pathological classification in national health records. This finding highlights the urgent need to improve diagnostic capacity, histopathological training, and disease coding practices in Colombia, in order to strengthen mesothelioma surveillance and guide national health strategies [16,26].

**Figure 4:**
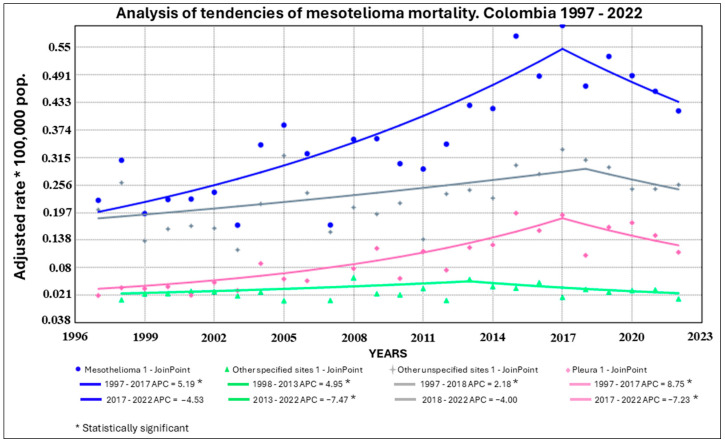
Trend analysis of mesothelioma mortality and diagnosis types, Colombia, 1997–2022. Source: Based on data compiled by the authors.

Age- and sex-adjusted mortality rates were calculated for each mesothelioma subtype throughout the study period. The lowest national mortality rates were observed in 2003 and 2007, both with 0.17 deaths per 100,000 inhabitants, while the highest rate was reported in 2017, reaching 0.59 per 100,000 inhabitants (Figure 4). This variation reflects both epidemiological trends and possible improvements in disease registration and diagnosis over time.

An upward trend in mesothelioma mortality rates was observed in Colombia starting in 2012, with the study period closing in 2022 at a rate of 0.41 deaths per 100,000 inhabitants. However, a statistically significant decline in the Annual Percent Change (APC) of −4.53% was recorded between 2017 and 2022, suggesting a downward trend during the final years of the study (Figure 4).

When analyzed by diagnosis type, several statistically significant increases were observed over time: mesothelioma of other specified sites (ICD-10: C457) showed an APC of +2.51% between 1997 and 2018; mesothelioma of specified sites increased by +4.95% from 1997 to 2013; and pleural mesothelioma (C450) exhibited the steepest rise, with an APC of +8.75% from 1997 to 2017. Following these periods of increase, a decline in all mesothelioma types was observed. This decline coincides with the COVID-19 pandemic, a period that may have affected the accuracy and classification of causes of death in national mortality records [27,28].

**Figure 5:**
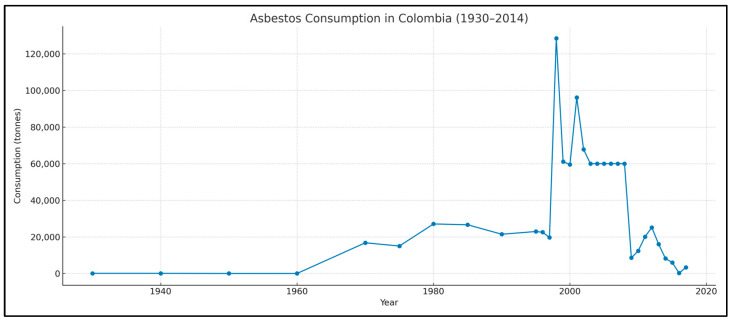
Historical asbestos consumption in Colombia (tonnes), 1960–2017. Source: United States Geological Survey.

The long history of industrial asbestos use in Colombia provides important background for interpreting these mortality trends. As shown in Figure 5, national asbestos consumption increased dramatically from the 1960s through the early 2000s, peaking above 120,000 tonnes per year. These high levels of use—sustained over decades—align with the long latency of mesothelioma and help explain the temporal distribution of cases in recent years.

**Figure 6:**
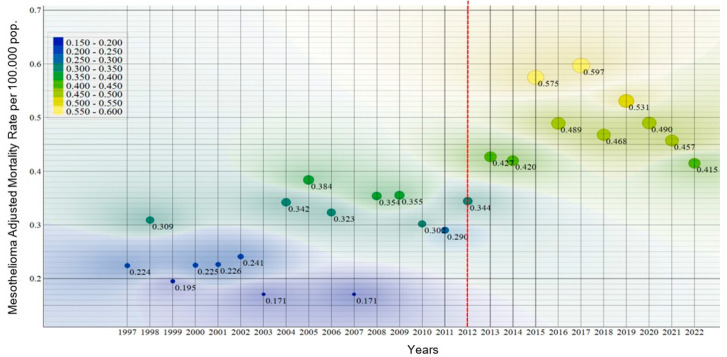
Nested dispersion of adjusted mesothelioma mortality rates, Colombia, 1997–2022. Source: Based on data compiled by the authors.

The nested dispersion analysis of age- and sex-adjusted mesothelioma mortality rates reveals distinct temporal patterns (Figure 6). In the color scale applied, blue corresponds to the lowest mortality rates, ranging from 0.224 to 0.350, predominantly observed during the period 1997 to 2012, with minor fluctuations in 2005 (0.384), 2008 (0.354), and 2009 (0.355). A transition to green tones, indicating intermediate rates, is observed in 2012 (0.424) and 2013 (0.420), and again in 2022 (0.415). The highest rates, highlighted in yellow, were recorded in 2015 (0.575) and 2017 (0.597).

The observed shift in mesothelioma mortality over time reveals a clear distinction between the periods 1997–2012 and 2013–2022. This temporal break may reflect the emergence of two distinct population segments, shaped by variations in exposure history, disease latency, and diagnostic capacity across decades.

The rise in mortality after 2011 is plausibly linked to the cumulative effect of past asbestos exposures, given the typical latency period of 20 to 40 years. Colombia’s industrial use of asbestos began in 1942 with the establishment of the Eternit plant in Sibaté (near Bogotá) and expanded significantly in the following decades. Additionally, increased diagnostic awareness, improved access to tools such as immunohistochemistry, and advances in mortality reporting systems may have contributed to greater case detection in more recent years.

**Figure 7:**
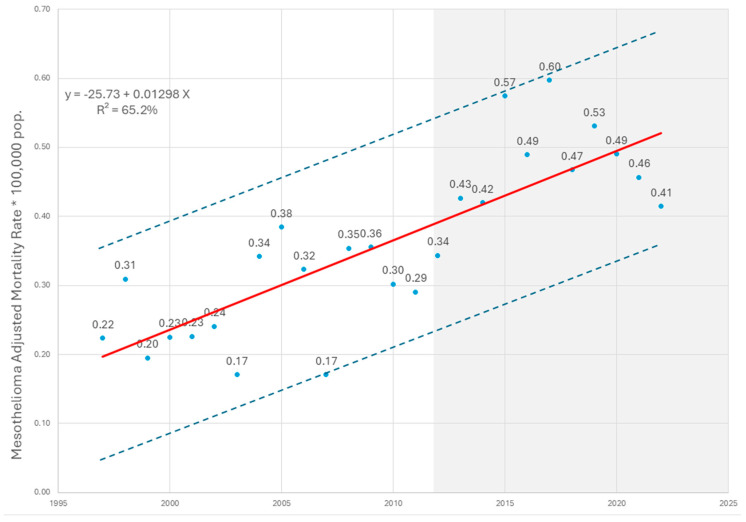
Process control chart of median mesothelioma mortality, Colombia, 1997–2022. Source: Based on data compiled by the authors.

A dispersion analysis using a process control chart was performed to examine the behavior of the median of adjusted mesothelioma mortality rates over the study period (Figure 7). The results show a sustained upward trend in the median rate, with a notable deviation during the final years (2020 and 2021), corresponding to the COVID-19 pandemic—a period marked by anomalies in mortality notification and classification across health systems [27,28].

The coefficient of determination (R^2^) calculated for this trend indicates that 65.2% of the variation in mesothelioma mortality can be explained by the time variable, underscoring the temporal association and potential cumulative effect of historical asbestos exposure in the Colombian population.

**Figure 8:**
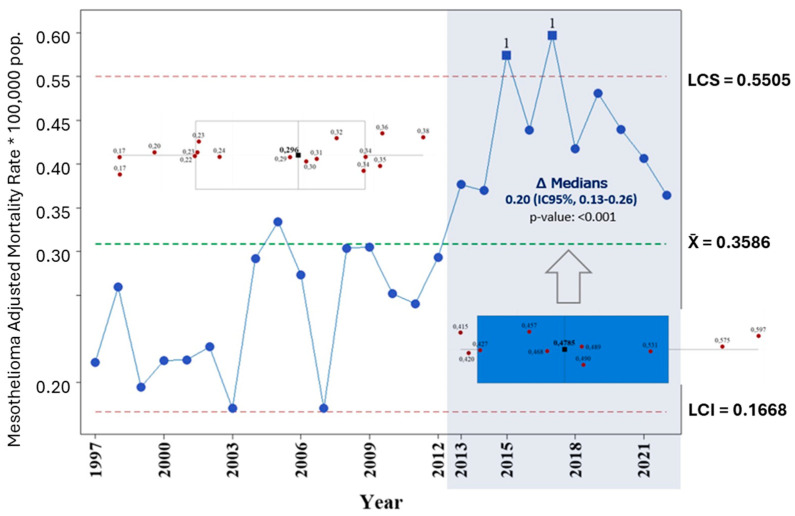
Difference in median mesothelioma mortality rates, 1997–2012 vs. 2013–2022. Source: Based on data compiled by the authors. Blue dots represent annual adjusted mortality rates per 100,000 population. The control limits (LCS and LCI) and the average line ($\bar{X}$) are shown for reference. Box plots summarize the distribution of mortality rates for the periods 1997–2012 and 2013–2022. Square symbols indicate the median values for each period. The statistically significant difference between the two medians (*p* < 0.001; 95% CI: 0.13–0.26) was determined using the Mann–Whitney U test. Values marked with “1” correspond to years in which the adjusted mortality rate exceeded the upper control limit predicted by the model.

A comparative analysis of median adjusted mortality rates was conducted for two distinct periods—1997–2012 and 2013–2022—using the Mann–Whitney U test. The median rate for the first period was 0.296, while for the second it increased to 0.4785, with a difference of 0.20 (95% CI: 0.13 to 0.26; *p* < 0.001), indicating a statistically significant difference between the two temporal segments (Figure 8).

These findings support the hypothesis of a structural shift in mesothelioma mortality over time, potentially reflecting changes in exposure patterns, latency periods, or diagnostic and reporting practices between the two decades.

The annual number of mesothelioma cases diagnosed and treated in Colombia, as reported in SISPRO (Individual Service Provision Records), has been available since 2009. The highest number of reported cases occurred in 2014 (n = 1492), followed by 2019 (n = 1015) and 2020 (n = 829). However, these morbidity figures should not be directly compared with the 1539 mesothelioma deaths recorded by DANE over the full 25-year period, as the two databases reflect different dimensions of the disease: SISPRO registers diagnosed and treated cases (morbidity), while DANE records mortality.

**Figure 9:**
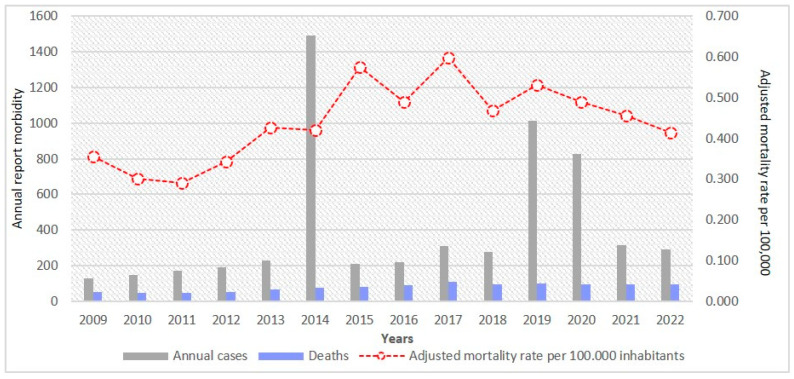
Annual mesothelioma cases, deaths, and adjusted mortality rate, Colombia, 2009–2022. Source: National Administrative Department of Statistics. Comprehensive Social Protection Information System (SISPRO).

Moreover, it was not possible to determine how many of the SISPRO-reported cases ultimately resulted in death, as no linkage mechanism currently exists between the two datasets. These discrepancies do not necessarily indicate data errors but instead highlight persistent issues of under-recording and fragmentation within Colombia’s health information systems. Such limitations undermine comprehensive epidemiological surveillance and emphasize the need for interoperability between morbidity and mortality data for asbestos-related diseases. This data divergence is illustrated in Figure 9, where SISPRO-reported morbidity is displayed alongside adjusted mesothelioma mortality rates.

The temporal overlap between peaks in mesothelioma incidence (2016–2017) and mortality (2015–2017) in Colombia is particularly notable, even though they reflect distinct dimensions of disease measurement. This convergence occurred prior to the 2019 asbestos ban and does not correspond to any documented changes in national reporting systems. Further investigation is warranted to assess whether the pattern reflects variations in actual exposure, changes in case detection, or other contextual influences.

The occupation variable began to be systematically recorded in Colombia in 2008, although with a low degree of completeness. According to the nominal mortality data, 39.8% of mesothelioma deaths lacked occupation information. Among the cases with available data, 23.1% were recorded as household-related occupations (primarily homemakers), followed by retired individuals (6.8%), and officials, workers, artisans, and related trades (5.6%) (Table 1).

Among the reported occupations of individuals who died from mesothelioma in Colombia, a significant proportion were categorized under “elementary occupations”, a group that includes manual laborers, construction workers, cleaners, and other low-skilled jobs involving routine physical tasks. This finding is particularly relevant given that many of these occupations are historically linked to high levels of asbestos exposure, especially in sectors such as construction, manufacturing, and mining, where asbestos was extensively used.

**Figure 10:**
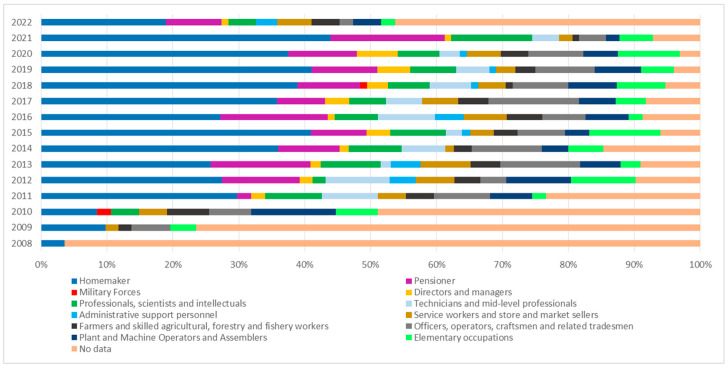
Annual proportion of mesothelioma cases by occupation type, Colombia, 2008–2022. Source: DANE—National Administrative Department of Statistics.

Over time, an increase in mesothelioma cases has also been observed among individuals classified as professionals, scientists, intellectuals, as well as mid-level technicians and professionals. Meanwhile, less frequent occupational categories include military personnel, administrative support staff, and students (Figure 10).

In Colombia, 93.9% of departments (n = 31) reported at least one death from mesothelioma during the 25-year study period. However, departments such as Amazonas and San Andrés registered no mesothelioma-related deaths. In contrast, Bogotá and Valle del Cauca reported cases in every year of the analysis period, indicating sustained patterns of reported mortality in these regions.

When analyzing the distribution of deaths by place of residence, Bogotá accounted for the highest proportion, with 43% (n = 666) of all reported cases, followed by Cundinamarca with 16% (n = 241) and Antioquia with 8% (n = 116). At the opposite end of the spectrum, departments such as Vaupés, Caquetá, Guaviare, and Vichada reported the lowest frequencies, with only two or three deaths each over the entire 25-year period (Table 2).

Between 1997 and 2022, mesothelioma deaths were reported in 238 cities across Colombia, representing 21.5% of all municipalities when analyzed by place of residence. In the analysis of adjusted mortality rates by year and city, the highest rates were observed in Beltrán (Cundinamarca) in 2012 and Chamezá (Casanare) in 2015, each reporting 215 deaths per 100,000 inhabitants. Notably, no deaths were recorded in these cities during other years of the study period.

Approximately 20 cities, located in Santander, Boyacá, Antioquia, Huila, Casanare, and Cundinamarca, reported adjusted rates above 40 deaths per 100,000 inhabitants in one to three isolated years.

The cities with the highest number of years reporting mesothelioma deaths were Bogotá, Medellín, Soacha, Cali, Sibaté, and Bucaramanga (Table 3). Among these, Sibaté stands out not only for having reported mesothelioma deaths in 18 of the 25 years but also for presenting the highest sustained adjusted mortality rates, ranging from 4.8 to 38.36 deaths per 100,000 inhabitants, which aligns with its historical role as a major center of asbestos production [7].

**Figure 11:**
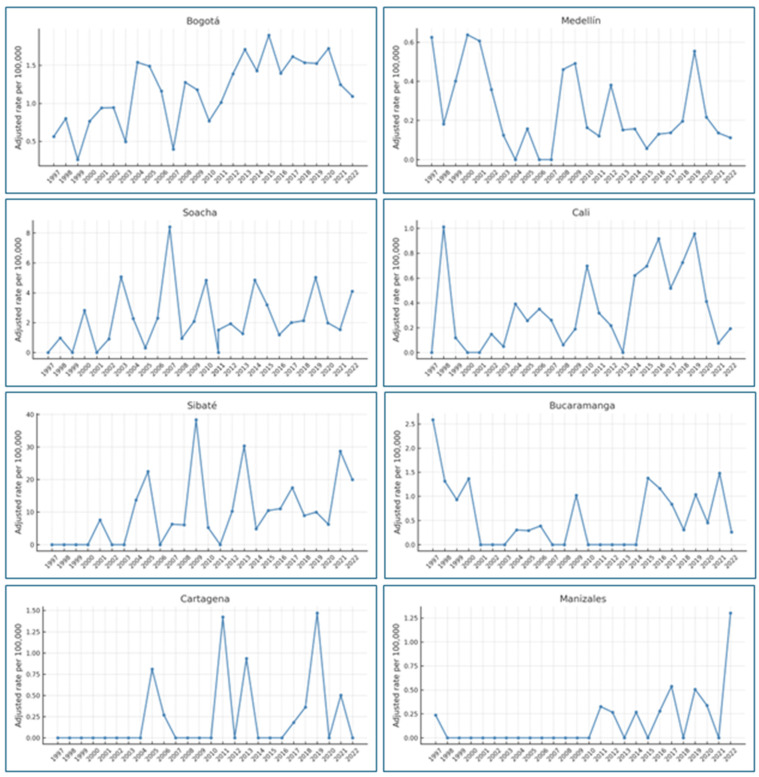
Adjusted mesothelioma mortality in asbestos-exposed cities, Colombia, 1997–2022. Source: Based on data compiled by the authors.

In addition to historically industrial cities, the analysis included locations not traditionally associated with asbestos production. For example, Manizales, a city with known historical exposure, and Cartagena, where such exposure is not widely documented, both exhibited fluctuating mesothelioma mortality rates over the study period. Cartagena recorded its highest adjusted mortality rate in 2019, with 1.46 deaths per 100,000 inhabitants, while Manizales peaked in 2022 with 1.30 deaths per 100,000 inhabitants (Figure 11).

It is important to note that Colombia lacks specialized centers for the diagnosis and treatment of asbestos-related diseases. Instead, mesothelioma patients are typically managed through general oncology services within hospitals that handle high-cost conditions. This institutional arrangement may influence the geographic distribution of reported cases, as patients often seek diagnosis and treatment in major urban centers, rather than in the regions where exposure actually occurred.

In Colombia, the diagnosis of mesothelioma typically relies on a combination of clinical, radiological, and pathological tools. Imaging studies such as chest X-rays and computed tomography (CT) are commonly used to identify pleural abnormalities. However, in practice, definitive diagnosis almost invariably involves histopathological examination supported by immunohistochemistry (IHC). This approach aligns with international diagnostic standards. Although access to these tools may be limited in some regions, IHC is routinely used in confirmed mesothelioma cases, particularly in specialized medical centers.

**Figure 12:**
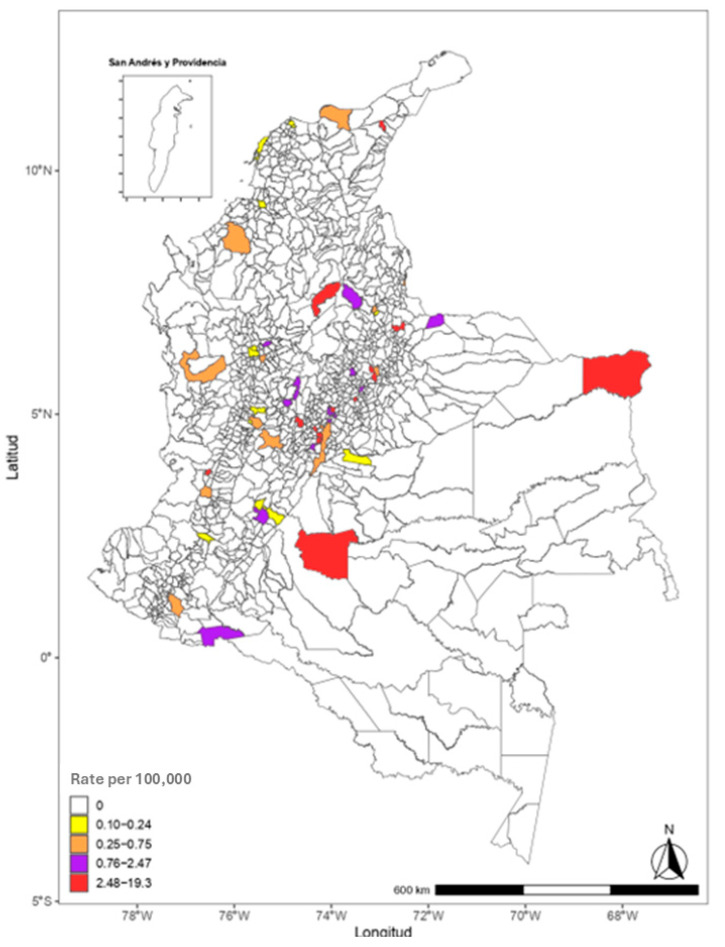
Mortality rate in adults aged 45–59 years, Colombia, 2013–2022. Source: Based on data compiled by the authors.

An analysis of accumulated mesothelioma mortality over the last ten years, disaggregated by age groups, revealed that in 51 cities, deaths occurred among adults aged 45 to 59 years. The highest quartile of adjusted mortality rates in this age group was observed in the following cities, listed in descending order: Sibaté (Cundinamarca), Concepción (Santander), Turmequé (Boyacá), Distracción (La Guajira), San Juan de Rioseco (Cundinamarca), Bojacá (Cundinamarca), Puerto Carreño (Vichada), Restrepo (Valle del Cauca), Paipa (Boyacá), La Macarena (Meta), Soacha (Cundinamarca), San Pablo (Bolívar), and Cogua (Cundinamarca) (Figure 12).

These findings highlight a concerning pattern of premature mortality from a preventable disease, particularly in small and medium-sized municipalities, many of which are located in historically exposed or underserved regions.

## 4. Discussion

Mesothelioma is a rare and aggressive malignancy with high lethality and poor prognosis [1,2,3,4]. Over the past six decades, histopathological, epidemiological, and occupational studies have consistently established mesothelioma as the most specific indicator of asbestos exposure, even more so than lung cancer [29]. In Colombia, the disease is considered to have a monocausal origin, with estimates indicating that more than 90% of cases are attributable to asbestos exposure. Furthermore, it is well established that all types of asbestos fibers are capable of causing mesothelioma in humans [17,30].

There are historical records of asbestos imports to Colombia dating back to 1930, with estimates of approximately 77 tons [31] (Figure 5). Industrial use of asbestos began in 1942 with the introduction of the Eternit brand to the national market. That same year, the first asbestos processing plant was established in Sibaté, near Bogotá, under the name Eternit Colombiana [7].

Subsequently, in 1945, subsidiaries were opened in Barranquilla (Eternit Atlántico) and Yumbo (Eternit Pacífico). In 1957, the Chaid Neme Hermanos business group began using asbestos in the manufacture of automotive parts and established Indubestos in Bogotá, a plant specialized in producing brake components and gaskets. Later, in Manizales, two additional companies were founded: Colombit in 1967 and Manilit in 1983 [7].

The broad territorial distribution of asbestos-related industries throughout Colombia likely generated decades-long environmental and occupational exposure risks, not only for workers but also for surrounding communities and informal asbestos traders.

Although the country historically relied on imports, a chrysotile (white asbestos) mine operated for several years in the municipality of Campamento, Antioquia. In addition, crocidolite was used alongside chrysotile in some industrial applications, including asbestos-cement roofing products. The use of crocidolite was formally prohibited in 1998 through Law 436, which approved ILO Convention 162 on the safe use of asbestos [32]. Later, Law 1968 of 2019 established a complete ban on all types of asbestos in Colombia, including chrysotile [8]. This toxic legacy remains embedded in the built environment of many urban and rural areas, posing ongoing risks. Although Law 1968 mandates the development of a national policy for managing installed asbestos, the Colombian government has yet to design and implement a comprehensive framework for its identification, safe removal, final disposal, and long-term control.

In Colombia, a total of 1539 deaths from mesothelioma were recorded during the study period, a figure that aligns with global estimates from the Global Burden of Disease (GBD) study [33]. According to our analysis, the male-to-female mesothelioma mortality ratio in Colombia was 2:1, contrasting with the global average of 3:1. This relatively higher burden in women may be explained by non-occupational exposure routes, including handling and laundering asbestos-contaminated clothing from family members who worked in asbestos-related industries [34]. In addition, the use of talcum powder contaminated with asbestos has been identified as a plausible risk factor for mesothelioma among women [25]. These para-occupational and environmental exposures are often under-recognized in national health surveillance systems, yet may represent a significant share of the disease burden.

Trend analysis of age- and sex-adjusted mesothelioma mortality rates revealed a statistically significant decline across all mesothelioma types between 2017 or 2018 and 2022. However, this apparent reduction is likely to be artefactual, coinciding with the COVID-19 pandemic, which disrupted the classification and notification of causes of death. During this period, individuals with pre-existing chronic or respiratory diseases—such as mesothelioma—may have had their deaths recorded as COVID-19-related, due to changes in diagnostic practices, corpse management protocols, and a general decrease in community transmission reporting [27,28].

Data from Colombia indicate that 27.6% of mesothelioma deaths were attributed to the pleura, while the most frequent diagnostic category was mesothelioma of unspecified site, accounting for 61.3% of cases. This may reflect diagnostic limitations at the hospital level, including lack of awareness among physicians regarding the various ICD-10 codes for mesothelioma, late-stage diagnoses, absence of histopathological confirmation, or inadequate biopsy samples, particularly in cases where the disease had already metastasized [26,35].

Approximately 62% of mesothelioma deaths occurred in individuals over 60 years of age, many of whom were recorded as pensioners or homemakers at the time of death. Unfortunately, it was not possible to reconstruct prior occupational histories in these cases to determine the precise sources of exposure. Some studies suggest that the risk of cancer-related mortality, including mesothelioma, decreases when the first occupational exposure occurs after the age of 50 [36]. Nevertheless, cumulative asbestos exposure—whether direct or indirect—remains the primary cause of mesothelioma worldwide [30].

Meanwhile, 38% of mesothelioma deaths occurred in individuals under 60 years of age, pointing to the possibility of early-onset exposure through multiple routes, including construction materials, contaminated water tanks, and inadequate asbestos waste disposal [7]. Other plausible sources may include household exposure or the use of asbestos-contaminated talc [25].

These patterns are also reflected in the cities with the highest and most sustained mesothelioma mortality rates, including Sibaté, Bogotá, Soacha, Medellín, Manizales, Cali, and Bucaramanga. While some of these cities are directly linked to asbestos-related industries or mining activities, others may have been affected through residential proximity to production centers, para-occupational exposure, or regional labor migration patterns.

Beyond Sibaté, several other cities with elevated mesothelioma mortality rates—such as Soacha, Bogotá, Medellín, Manizales, Cali, and Bucaramanga—present distinct contextual factors that may explain these patterns. Soacha is directly adjacent to Sibaté and likely served as a residential hub for workers employed at the Eternit asbestos plant, exposing families to para-occupational and environmental risk. Medellín is the capital of Antioquia, the department where Colombia’s only chrysotile mine operated, potentially exposing both miners and surrounding populations. Manizales hosted two asbestos-cement plants, and the area near Cali was home to another major industrial facility of the same kind. In the case of Bucaramanga, although there is no record of direct industrial asbestos activity, further study is needed to assess the impact of intermunicipal labor migration and regional exposure pathways. Notably, Bucaramanga—the capital of the department of Santander—shares regional ties with Barrancabermeja, the department’s second largest city and the site of Colombia’s largest oil refinery, a sector known internationally for historical asbestos use.

Although this study utilized the most complete national mortality data available, it was not possible to accurately identify key institutional variables—such as the health facilities where cases were treated, or the Health Promotion Entities (EPS) and Occupational Risk Administrators (ARL) responsible for follow-up and management. This constitutes a significant limitation for the prioritization of health sector interventions.

Although Colombia has existing frameworks and records related to occupational disease surveillance, including programs implemented by the ARLs, studies suggest that the full implementation of the Occupational Health and Safety Management System (SG-SST) remains limited, particularly in tracking long-latency diseases such as mesothelioma [37,38].

In addition, it is essential to implement actions aimed at reducing the risk of occupational, domestic, and environmental asbestos exposure, particularly within family and community settings.

Continued research on mesothelioma and other asbestos-related diseases is necessary to support and strengthen regulatory frameworks, especially those stemming from the law that banned asbestos in Colombia [8]. This includes advancing efforts toward the complete substitution of asbestos-containing materials, and addressing current sources of exposure—whether in homes, workplaces, or environmental contexts—in order to ensure the effective implementation of a national program for the elimination of asbestos-related diseases.

To this end, the creation of a national mesothelioma registry with legal and notarial support would be a valuable tool for surveillance and policymaking. Additionally, there is a pressing need to improve training for pathologists, enabling them to recognize mesothelioma more accurately, and for pulmonologists and radiologists to enhance their ability to identify the disease through clinical and imaging interpretation.

The proposal to establish a national mesothelioma registry in Colombia draws inspiration from international models that have proven effective in supporting public health surveillance, epidemiological research, and compensation systems. Countries such as Italy (ReNaM) [39], Australia (AMR) [40], and the United Kingdom [41] maintain mesothelioma registries that integrate mortality records, occupational history, and exposure data. These systems have contributed significantly to understanding disease patterns and the development of prevention policies. A similar registry in Colombia would help close the current gap between morbidity and mortality data and support intersectoral action to address the asbestos legacy.

In a 2024 presentation on the asbestos-related disease burden in Colombia, Dr. Tim Driscoll, member of the GBD occupational health team, highlighted key methodological limitations of the global model. These include the reliance on risk estimates primarily derived from high-income countries, the use of blunt exposure metrics, and the exclusion of informal and domestic labor—factors that are especially relevant in Latin American contexts. Importantly, the GBD framework itself acknowledges that mesothelioma rates are likely moderately under-estimated, reinforcing the relevance of detailed national mortality analyses such as the one conducted in this study [42].

## 5. Conclusions

This study provides the most comprehensive analysis to date of mesothelioma mortality in Colombia, documenting 1539 deaths between 1997 and 2022. The results confirm a sustained and geographically widespread burden, with significant impacts among men and women, particularly in industrial and urban areas historically associated with asbestos exposure.

The analysis reveals a consistent pattern of underreporting and diagnostic imprecision, especially in the high proportion of cases classified as mesothelioma of unspecified site. These findings underscore the urgent need to strengthen diagnostic capacities, including histopathology and imaging interpretation, and to improve disease registration and ICD-10 coding practices.

A key contribution of this work lies in the use of official mortality data (DANE) and case-by-case review, offering a direct national estimate that complements and contrasts with global models. While the Global Burden of Disease (GBD) study estimated 98 mesothelioma deaths in Colombia for 2021, our study identifies an annual average of approximately 62 deaths, based on individual death records. Both estimates provide an indication of the actual number of deaths. The difference highlights the value of using different approaches—statistical modeling from available national datasets and regional estimates in the case of the GBD, and detailed analyses of primary mortality records, as presented in this study—to inform public health decisions.

These findings support the establishment of a national mesothelioma registry, structured through legal and intersectoral mechanisms. They also underscore the need for sustained investment in occupational and environmental surveillance, the training of health professionals, and the implementation of policies that ensure prevention, compensation, and comprehensive care for populations affected by asbestos exposure in Colombia.

## Figures and Tables

**Table 1:** Reported occupations of people who died from mesothelioma, Colombia, 1997–2022.

Occupation	Deaths	Proportion
**Housewife (Para occupational)**	356	23.10%
**Pensioner**	105	6.80%
**Officers, laborers, craftsmen and related trades**	86	5.60%
**Professionals, scientists and intellectuals**	73	4.70%
**Plant and machine operators and assemblers**	61	4.00%
**Elementary occupations**	61	4.00%
**Technicians and mid-level professionals**	49	3.20%
**Service workers and store and market salespersons**	48	3.10%
**Farmers and skilled agricultural, forestry and fishery workers**	39	2.50%
**Directors and Managers**	28	1.80%
**Administrative support personnel**	16	1.00%
**Students**	3	0.20%
**Military Forces**	2	0.10%
**No data**	612	39.80%
**Total**	1539	100%

Source: DANE—National Administrative Department of Statistics.

**Table 2:** Absolute frequency of mesothelioma deaths by Department of residence, Colombia, 1997–2022.

Territory	97	98	99	0	1	2	3	4	5	6	7	8	9	10	11	12	13	14	15	16	17	18	19	20	21	22
Bogotá	7	11	9	13	17	17	13	23	21	23	11	22	21	18	21	27	37	34	36	35	40	40	42	49	43	36
Cundinamarca		5	4	4	2	4	6	8	8	3	5	7	11	10	6	8	9	12	14	13	17	15	18	15	13	24
Antioquia	6	4	3	5	5	3	2	1	5	5		5	4	3	3	3	4	6	5	4	4	5	9	7	9	6
Valle del Cauca	1	8	3	2	1	2	1	7	4	1	3	2	1	7	2	1	2	6	7	11	7	11	7	6	7	4
Santander	2	4	3	1	1	3	1	1	1	1	1	1	1		1		3		4	4	5	5	5	4	7	2
Boyacá			2	1	2		3	1	2	1		2		1		1	2	2	3	2	5	4	2	2	2	4
Nariño			1	2				2			1	1	1		2	1	1	2	2	2	7	1	1		1	
Tolima		1	2	1	1			1	1			1		1		3		1	2		5	3		2		2
Atlántico	1	1	1		1						2			1		1	1	2	2	1	3		2	2		3
Huila			2			1		1		1	1	1	2			4		1	1	1			1	3	2	1
Magdalena		1		1		2				1				1	1			1		3	3		2	2	2	2
Bolivar									1	1		1			3	1	1		1		1	2	4		3	2
Caldas	1														3	1	1	2		3	1		3	2	1	3
Meta		1		1	1				2	1		2	2					3		2	1	3			1	
Norte de Santander						2	1						2	1			1		2	2	3	1		1	1	1
Risaralda												4	1	1	1		2	1		3	1	1				2
Cauca			1				1					3	2	1			1			2	2		1			
Quindío	1			1			2			1		1	1		1				1	3			1		1	
Córdoba							1		1										2		1	2			1	
Cesar					1								1	1	1			1			1		1			
Casanare								1						1					1	1	1				1	
Arauca					1							1												1	1	
Chocó			1														1								1	
La Guajira												1														2
Sucre															1							1				1
Putumayo									1				1					1								
Vaupés	1																						1			
Caquetá												1														
Guaviare																									1	
Vichada																						1				
No data	4														1						1					
	24	36	32	32	33	34	31	46	47	39	24	56	51	47	47	51	66	75	83	92	109	95	100	96	98	95

Source: DANE—National Administrative Department of Statistics.

**Table 3:** Cities with the highest frequency in years of mesothelioma mortality, Colombia, 1997–2022.

City	Number of Years with Mortality	Minimum Rate	Maximum Rate
Bogotá	26	0.25	1.86
Medellín	23	0.05	0.63
Soacha	23	0.30	8.41
Cali	22	0.04	1.01
Sibaté	18	4.80	38.36
Bucaramanga	16	0.25	2.58

Source: DANE—National Administrative Department of Statistics.

## Data Availability

The data used in this study were obtained from the National Administrative Department of Statistics (DANE) and the Social Protection Health Information System (SISPRO) of Colombia. These datasets are not publicly available due to institutional restrictions. However, anonymized data relevant to the study’s analysis can be shared upon reasonable request and approval from the authors.
